# The role of transcytosis in the blood-retina barrier: from pathophysiological functions to drug delivery

**DOI:** 10.3389/fphar.2025.1565382

**Published:** 2025-04-16

**Authors:** Chun-Lin Zhang, Jing-Jie Ma, Xiang Li, Hai-Qing Yan, Yong-Kun Gui, Zhi-Xin Yan, Ming-Feng You, Ping Zhang

**Affiliations:** ^1^ Department of Neurology, The First Affiliated Hospital of Xinxiang Medical University, Xinxiang, China; ^2^ Department of Audit, The First Affiliated Hospital of Xinxiang Medical University, Xinxiang, China; ^3^ Department of Neurology, The Affiliated Hospital of Guizhou Medical University, Guiyang, China

**Keywords:** transcytosis, blood-retina barrier, barrier function, vascular leakage, drug delivery

## Abstract

The blood-retina barrier (BRB) serves as a critical interface that separates the retina from the circulatory system, playing an essential role in preserving the homeostasis of the microenvironment within the retina. Specialized tight junctions and limited vesicle trafficking restrict paracellular and transcellular transport, respectively, thereby maintaining BRB barrier properties. Additionally, transcytosis of macromolecules through retinal vascular endothelial cells constitutes a primary mechanism for transporting substances from the vascular compartment into the surrounding tissue. This review summarizes the fundamental aspects of transcytosis including its function in the healthy retina, the biochemical properties of transcytosis, and the methodologies used to study this process. Furthermore, we discuss the current understanding of transcytosis in the context of pathological BRB breakdown and present recent findings that highlight significant advances in drug delivery to the retina based on transcytosis.

## 1 Introduction

The retina, an extension of the central nervous system (CNS), plays a crucial role in transforming the image projected on the surface of retina into nerve impulses, facilitating the process of visual perception. The functional retina relies on a stable homeostatic neurovascular microenvironment that facilitates the effective and efficient transportation of oxygen and nutrient from the blood, expels metabolic wastes and protects from pathogens and blood-borne toxins. Previous studies indicate that the strict regulation of solutes, fluids, and cells crossing the BRB is accomplished through two well-defined cell junctions: adherens junctions (AJs) and tight junctions (TJs) (Campbell and Humphries, 2012). Continuous expression of these junction proteins along the basal or apical perimeter of cells establishes a characteristic sealant ring, which restricts the substance exchange through the paracellular pathway (Peng et al., 2003). Besides specialized tight junction complexes, retinal endothelial cells have another property that is essential for their barrier function: a low rate of transcytosis for limiting transcellular transport. The role of TJs and AJs in retinal vascular barrier function is well studied, while the role of endothelial transcytosis in the permeability of the BRB has recently been identified (Ben-Zvi et al., 2014; Knowland et al., 2014; Greene and Campbell, 2016). The breakdown of the BRB especially is a hallmark of numerous retinal degenerative diseases, including diabetic retinopathy (DR), cystoid macular edema, age-related macular degeneration (AMD), and glaucoma (Cunha-Vaz et al., 2011). Recent researches have highlighted the significant contribution of enhanced endothelial transcytosis to the development of retinal edema (Klaassen et al., 2013; Wang et al., 2020; Zhang et al., 2021).

Transcytosis, also known as transcellular vesicular transport, refers to the intracellular transportation of large molecules (>10 kDa) within vesicles through endothelial cells or epithelial cells (Tuma and Hubbard, 2003; Mehta and Malik, 2006; Garcia-Castillo et al., 2017; Fung et al., 2018). This process has been observed in multiple organs such as the lung, brain, intestine, heart, and retina (Bendayan and Rasio, 1996; Oh et al., 2007; Zhou et al., 2015; Andreone et al., 2017; Chow and Gu, 2017). Multiple macromolecular cargo is conveyed through transcytosis, including low-density lipoproteins, albumin, immunoglobulins, lipids, and insulin (Lee and Klip, 2016; Li et al., 2017; Huang et al., 2019; Raheel et al., 2019; Stapleton et al., 2019). Researches about transcytosis in the CNS barrier function mainly focus on the BBB, while the studies about transcytosis in the BRB are relatively lagging behind. At 1986, E Essner et al. studied the surface-associated vesicles in retinal vessels by intravitreal injection of lactoperoxidase or peroxidase (Essner et al., 1986). Their preliminary study indicated that the vesicles were located along the abluminal surface of the vascular endothelial cells. Gregory W Thomas et al. found that 5% human serum albumin reduced transcytosis in human retinal endothelial cells (HREC), which may affect HREC permeability (Thomas et al., 2016). The advancement of electron microscopy technology has significantly accelerated the investigation of the ultra-microstructure of transcellular vesicles in retinal vascular endothelial cells. Leakage from immature retinal vessels occurs exclusively through transcytosis, despite the establishment of functional tight junctions when the vessels first grow into the CNS (Chow and Gu, 2017). The spatio-temporal regulation of transcytosis is crucial for the development of a functional BRB, and major facilitator superfamily domain containing 2a (Mfsd2a) is critical in inhibiting transcytosis at the BRB (Chow and Gu, 2017).

Recent studies have revealed the diverse functions of transcytosis in the BRB. In this review, we summarize the function of transcytosis in the healthy retina, offer a concise overview of the biochemical properties of transcytosis and the methodologies used to study this process. We then discuss the role of transcytosis in pathological BRB breakdown, and strategies for delivering drugs to the retina that leverage transcytosis mechanisms.

## 2 Transcytosis in healthy BRB

### 2.1 Endothelial transcytosis as a primary mechanism of transcellular transport across the BRB

**FIGURE 1:**
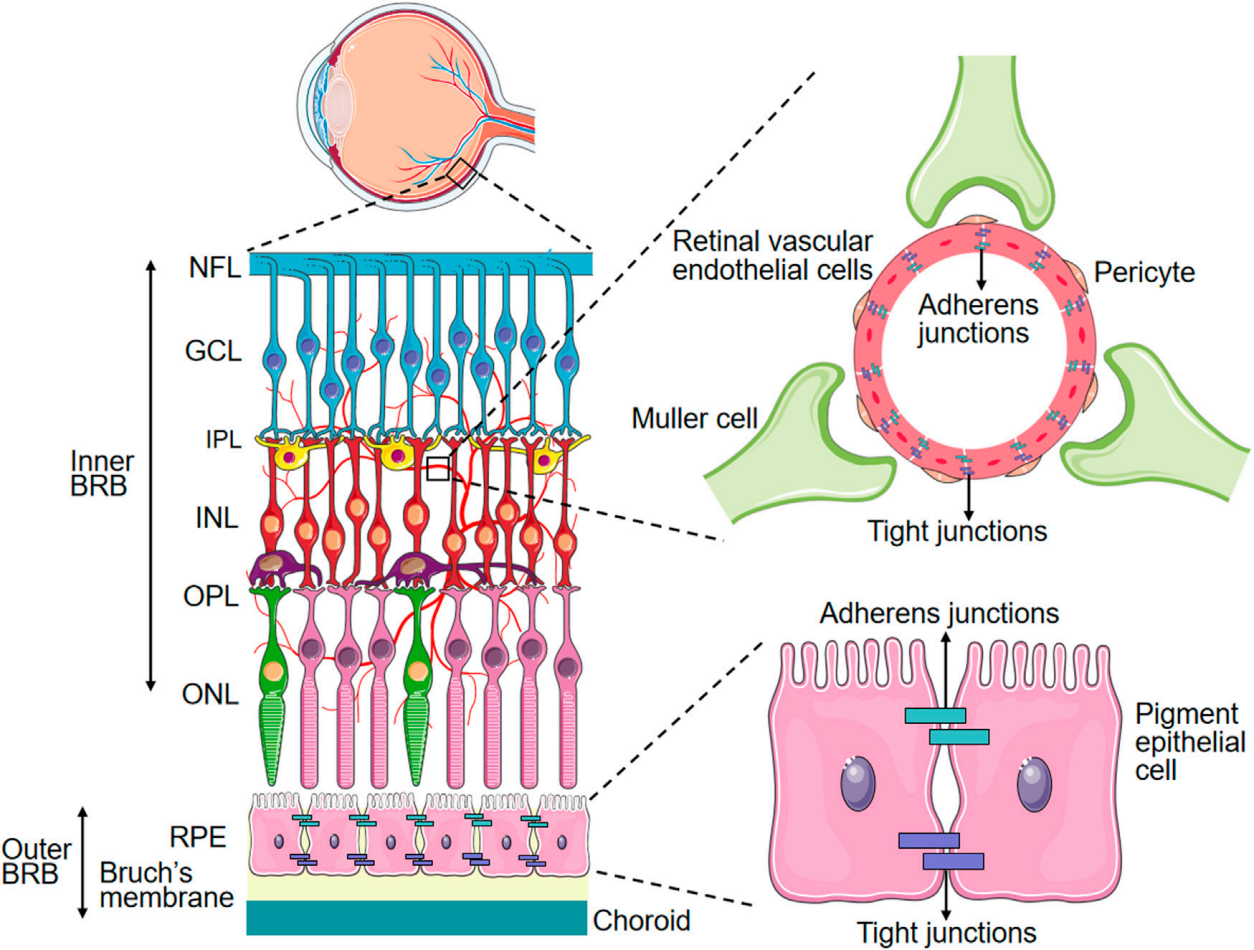
Schematic Representation of the BRB. PRE: Retinal pigment epithelium, ONL: Outer nuclear layer, OPL: Outer plexiform layer, INL: Inner nuclear layer, IPL: Inner plexiform layer, GCL: Ganglion cell layer, NFL: Nerve fiber layer.

Microvascular endothelial cells in the BRB exhibit a specialized barrier property distinct from that of peripheral endothelium. The inner BRB consists of endothelial cells anchored on the basement membrane, covered by Muller cells. These vascular components, together with pericytes, exert regulatory control over retinal endothelial cell function and inner BRB integrity. The outer BRB is composed of retinal pigment epithelium dells, which modulates the transport between the choroidal capillaries and the retina (Figure 1). Microvascular endothelial cells in the BRB exhibit a specialized barrier property distinct from that of peripheral endothelium. This barrier function in retinal microvascular endothelial cells (RMECs) is established through the continuous arrangement of intercellular tight junctions and the maintenance of extremely low rates of transcytosis, which together regulate both paracellular transport and transcellular transport of substances across the BRB on physiological conditions. While previous studies have extensively reviewed paracellular transport in the BRB, this paper will concentrate on transcellular transport in the BRB.

Energy-dependent membrane transporters and vesicular transport mechanisms play crucial roles in regulating the precise movement of solutes and fluids across the BRB. Energy-dependent transcellular transport across RMECs is categorized into five main groups: ion transport, active efflux transport, carrier-mediated transport, caveolae-mediated transport and receptor-mediated transport. For instance, ion transport systems control the flux of ions across RMECs and include several key members such as sodium-potassium-two chloride (Na^+^-K^+^-2Cl^-^) cotransporter (NKCC), sodium pump (Na^+^, K^+^-ATPase), chloride–bicarbonate exchanger, sodium–hydrogen exchanger and sodium–calcium exchanger (Vorbrodt, 1988; O'Donnell et al., 2006; Taylor et al., 2006). The active efflux transport systems mediate the movement of molecules from nervous tissue into the systemic circulation, involving certain solute carrier transporters and adenosine triphosphate-binding cassette efflux transporters, such as organic anion transporter 3, breast cancer resistance protein, and P-glycoprotein (Hermann and Bassetti, 2007; Tajima et al., 2022). The carrier-mediated transport systems are responsible for the influx of certain amino acids, glucose, vitamins and lactate across RMECs (Hawkins et al., 2006; Ohtsuki and Terasaki, 2007; Spector and Johanson, 2007). Caveolae-mediated transport systems are responsible for transporting large molecules like lipoproteins, albumin, and insulin across vascular RMECs. The receptor-mediated transport systems play a role in transporting large proteins and neuroactive peptides across vascular endothelial cells, such as immunoglobulin G and transferrin (Jefferies et al., 1984; Méresse et al., 1989; Zlokovic, 1995; Deane et al., 2005). It is also noteworthy that there can be overlap between caveolae-mediated transport and receptor-mediated transport when the receptors are situated on the caveolae membrane.

Transcytosis refers to the vesicular transport of large molecules between the luminal and abluminal sides of cells and occurs in numerous cell types, such as epithelial cells, endothelial cells, intestinal cells, and neurons (Pulgar, 2018). This biological process encompasses endocytosis (internalization), intracellular vesicular transport, and exocytosis (release) (Pulgar, 2018). Currently, the specific types of vesicles and mechanisms of transcytosis involved in this process remain unclear. However, it is widely accepted that two primary forms of transcytosis exist in endothelial cells of the CNS: receptor-mediated transcytosis and nonselective adsorptive transcytosis. A limited set of peptides and proteins are transported across the BBB via receptor-mediated transcytosis in the CNS, including iron-transferrin, insulin, and low-density lipoprotein (LDL)-cholesterol (Tuma and Hubbard, 2003). Nonselective adsorptive transcytosis mediates the entry of molecules through charged interactions between the cell membrane and the cargo. Various cationic proteins, such as albumin, avidin, and histone, traverse the CNS barrier through this nonselective mechanism (Pardridge et al., 1989; Pardridge and Boado, 1991). Three types of endocytic vesicles have been identified in CNS endothelial cells: caveolae, which is involved in nonselective adsorptive transcytosis and receptor trafficking; clathrin-coated pits, which is primarily responsible for most receptor-mediated transcytosis; and macropinocytotic vesicles (Mayor and Pagano, 2007). Compared with other organs or tissues, fully developed RMECs exhibit a lower transcytosis rate, which is essential for preserving their barrier properties under physiological conditions (Predescu et al., 2004; Anderson, 2008). Transcytosis facilitates the transport of macromolecules from the bloodstream into the retina through specific receptors localized on the vesicular membranes. The low rate of transcytosis in barrier endothelial cells may be attributed to a lower abundance of caveolae within the luminal membrane. In non-barrier endothelial cells, it is achieved by the decreased of albumin receptor expression, caveolin-1, and other associated molecules.

In summary, the active transport of large molecules across endothelial cells in the BRB predominantly occurs through transcellular transport under normal physiological conditions. This process is mediated by mechanisms such as receptor-mediated and caveolae-mediated transport pathway.

### 2.2 Mfsd2a and PLVAPs are markers for endothelial transcytosis

Mfsd2a, a member of the major facilitator superfamily, was initially identified as an orphan transporter. The Mfsd2a gene spans approximately 14.3 kb and comprises 13 introns and 14 exons. This gene is highly conserved, with an amino acid sequence that is approximately 85% identical between mice and humans (Eser Ocak et al., 2020). Mfsd2a is selectively expressed in both retinal and cerebral microvessels in the CNS, and plays important roles in both lipid transport and transcytosis (Wong and Silver, 2020). Unlike most other members of the major facilitator superfamily, which transport water-soluble ligands like amino acids and sugars, Mfsd2a is distinct in its ability to transport lipids, particularly lysophosphatidylcholine (LPC) with unsaturated fatty acyl chains (Wong and Silver, 2020). In the brain, Mfsd2a is associated with a reduction in caveolae microdomains within vascular endothelial cells and leads to a significant decrease in transcytosis, independently of TJs (Andreone et al., 2017). In the eye, the spatiotemporal expression of Mfsd2a is closely linked to the gradual suppression of transcytosis, which helps establish a functional BRB (Chow and Gu, 2017). Additionally, Mfsd2a is involved in the transport of docosahexaenoic acid (DHA) in the photoreceptors of retinal pigment epithelial cells (RPEs) in mice (Wong et al., 2016). These findings underscore the importance of Mfsd2a in preserving the barrier function of vascular endothelial cells in both the brain and retina under normal physiological conditions. Additionally, the expression of Mfsd2a can be utilized as an indicator of inhibited transcytosis in the BBB and BRB.

Plasmalemma vesicle-related proteins (PLVAP) is the only discovered molecular component of stomatal diaphragms (SDs) and fenestral diaphragms (FDs) (Stan et al., 1997; Stan et al., 1999). The diaphragms create cap-like structures which connect the opening of caveolae, trans-endothelial channels and fenestrae (Schlingemann et al., 1985; Rothberg et al., 1992; Stan, 2005). PLVAP is a type II integral membrane N-glycosylated glycoprotein with a molecular weight of 55–65 kDa, forming homodimers *in situ* and binding to heparin at physiological pH (Hnasko et al., 2002; Stan, 2004). The molecular structure of PLVAP consists of a large extracellular domain (380 amino acids), a single span transmembrane domain and a short intracellular domain (27 amino acids) (Stan et al., 1999; Stan, 2005). PLVAP is believed to plays a significant role in regulating vascular permeability and be linked to compromised barrier function in several retinal disorders in various retinal diseases, such as wet AMD, DR and retinopathy of prematurity (ROP) (Wisniewska-Kruk et al., 2014; Wisniewska-Kruk et al., 2016; Nakagami et al., 2019). Therefore, PLVAPs expression is considered as a marker for increased endothelial transcytosis.

## 3 Biochemical properties of transcytosis

**FIGURE 2:**
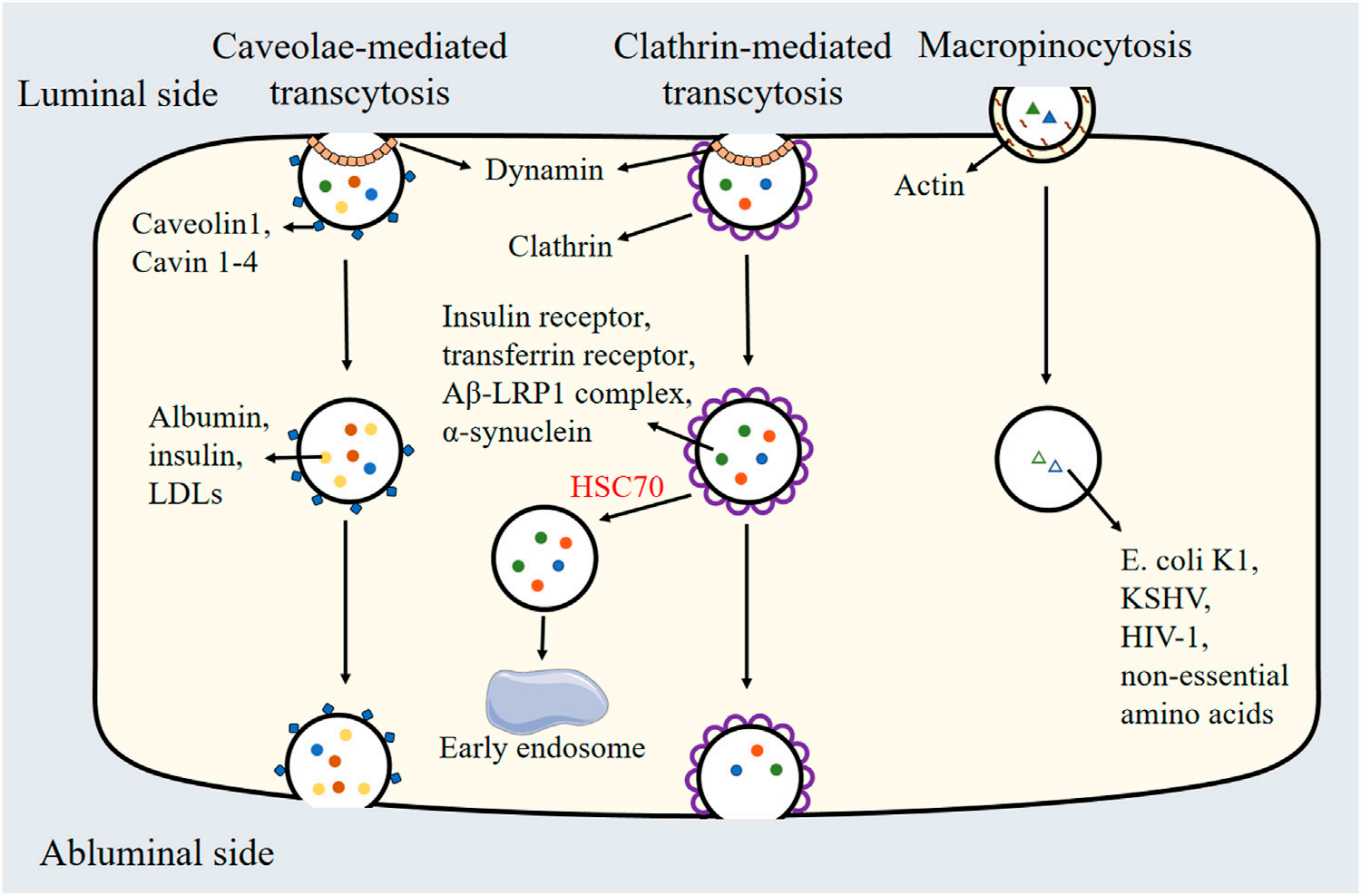
Schematic Representation of caveolae-mediated transcytosis, clathrin-mediated transcytosis, and micropinocytosis. LDLs: Low-density lipoproteins, HSC70: Heat shock cognate protein 70, Aβ: Amyloid-beta peptide, LRP1: Low-density lipoprotein receptor-related protein 1, *E. coli* K1: *Escherichia coli* K1, KSHV: Kaposi’s sarcoma-associated herpesvirus, HIV1: Human immunodeficiency virus 1.

Transcytosis in endothelial cells is regulated by distinct biochemical pathways. Caveolae-mediated transcytosis relies on cholesterol and sphingolipid-rich membrane domains stabilized by caveolin-1 and cavin proteins, enabling selective transport of macromolecules like albumin and LDLs. Clathrin-mediated transcytosis utilizes clathrin triskelions, adaptor proteins, and dynamin for receptor-specific cargo internalization and sorting. Macropinocytosis, driven by actin polymerization and signaling cascades, facilitates nonselective uptake of solutes via large vesicles, critical for nutrient scavenging and pathogen entry. These pathways are regulated by lipid composition, GTPases, and membrane-coating proteins, reflecting their unique biochemical architectures and functional roles in endothelial transport (Table 1 and Figure 2).

**TABLE 1:** Biochemical properties of caveolae-mediated transcytosis, clathrin-mediated transcytosis, and macropinocytosis.

Type	Mechanism	Structural features	Key molecules	Function/Characteristics	Examples
Caveolae-mediated transcytosis	Vesicle transport or transient trans-endothelial pores via fusion/fission	Bulb-shaped invaginations (40–80 nm), cholesterol/sphingolipid-rich, caveolin-1/cavin-coated, bipolar morphology	Caveolin-1, Cavin-1–4, dynamin	Selective transport of lipids and large molecules; stability depends on cholesterol and sphingolipid-rich membrane domains	Albumin, insulin, LDLs
Clathrin-mediated transcytosis	Clathrin-coated pits form vesicles *via* AP2/dynamin; HSC70 uncoats vesicles	Triskelion structures (clathrin heavy/light chains), AP2-coated pits, dynamin-mediated fission	Clathrin, AP2, dynamin, PICALM, HSC70, LRP1 receptor	Receptor-mediated endocytosis and transcytosis; selective cargo transport	Insulin receptor, transferrin receptor, Aβ-LRP1 complex, α-synuclein
Macropinocytosis	Actin-driven membrane ruffling forms large irregular vesicles	Irregular vesicles (200–500 nm); plasma membrane ruffling	Actin, MAPK signaling	Nonselective fluid/solute uptake; viral/bacterial entry	*E. coli* K1, KSHV, HIV-1, non-essential amino acids

LDLs, Low-density lipoproteins; AP2, Adaptor protein 2; PICALM, Phosphatidylinositol binding clathrin-assembly protein; HSC70: Heat shock cognate protein 70; Aβ, Amyloid-beta peptide; LRP1, Low-density lipoprotein receptor-related protein 1; MAPK, mitogen-activated protein kinase, *E. coli* K1: *Escherichia coli* K1; KSHV, Kaposi’s sarcoma-associated herpesvirus; HIV1, Human immunodeficiency virus 1.

### 3.1 Caveolae-mediated transcytosis

Caveolae are bulb-shaped plasma membrane invaginations and free cytoplasmic vesicles measuring between 40 and 80 nm in size (Parton, 2018). Although initially identified in heart endothelial cells, these vesicles are ubiquitous across various multiple types of cells, such as endothelial cells, adipocytes, fibroblasts, and smooth muscle cells (Palade, 1953; Pilch and Liu, 2011; Chettimada et al., 2015; Marsboom et al., 2017; Filippini et al., 2018). K.R. Peters et al. explored the distribution and structure of endothelial caveolae in cultured endothelial cells using scanning electron microscopy. Caveolae in endothelial cells exhibit flask-shaped invaginations (about 70–90 nm diameter) characterized by a distinctive striped bipolar surface architecture. High-resolution analysis reveals these vesicular structures possess 10-nm-wide meridional ridges separated by linear arrays of dimples. These ridges converge bidirectionally at diametrically opposed poles oriented parallel to the plasma membrane plane, forming a ‘bi-polar’ morphology distinct from other types of endothelial vesicles. Notably, the plasmalemma’s cytoplasmic surface between vesicles exhibited dense reticular organization, composed of irregularly arranged intermediate filaments (about 7–9 nm in diameter) (Peters et al., 1985). Although caveolae are abundant in multiple cells, their number and specific features vary among different cell types. For instance, caveolae density is highest in venular endothelium, reaching up to 1,200 caveolae/μm^3^ (Simionescu et al., 1974). Furthermore, endothelial cells possess a larger caveolar neck domain compared to fibroblasts (Richter et al., 2008). The caveolar neck domain recruit actin-binding proteins fission and GTPases, and the larger diameter in neck size may play a significant role in transcytosis. Cholesterol and sphingolipids are the primary components of caveolae, which are essential for their stability and formation (Lajoie and Nabi, 2010; Sengupta, 2012; Krishna and Sengupta, 2019). Depletion of membrane cholesterol disrupts caveolae stability, leading to loss of caveolae (Sengupta, 2012). Additionally, the reduction of phosphatidylserine levels diminishes caveola formation and dynamics, indicating that phospholipids are also functionally important (Hirama et al., 2017).

Caveolins are integral membrane proteins that are vital for various cellular functions, such as lipid trafficking, signal transduction, and endocytosis (Rothberg et al., 1992). Caveolin-1, the first discovered protein in caveolar vesicles in 1992, has significantly advanced our understanding of the function of caveolae, particularly within endothelial cells. This 22 kDa protein features a central hydrophobic domain that forms a hairpin structure within the membrane, with both its hydrophilic carboxyl and amino termini oriented towards the cytoplasm (Tang et al., 2021; Gokani and Bhatt, 2022; Puddu et al., 2023). Caveolin-1 is found in a variety of cell types, such as endothelial cells, adipocytes, and fibroblasts, and its α and β isoform expressed in varying proportions depending on the cell type (Fujimoto et al., 2000; Kogo et al., 2004). For example, lung endothelial cells primarily express the α isoform, while type I alveolar epithelial cells mainly express the β isoform. Notably, caveolin-1 is an essential component for caveolar biogenesis, it is also identified in non-caveolae membrane (Rothberg et al., 1992; Razani et al., 2001). Subsequent studies have indicated that caveolin-1 plays a key role in creating membrane curvature and sequestering cholesterol within caveolar membranes (Hoop et al., 2012). Interestingly, the expression pattern of caveolin-2 resembles that of caveolin-1. However, caveolin-2 is not unnecessary for caveolar formation (Vinten et al., 2005; Hill et al., 2008; Liu et al., 2008). Caveolin 3 is predominantly found in striated muscle and is required for caveolar formation. Interestingly, caveolin-1 alone is sufficient for caveolar morphogenesis, while caveolin-3 works in conjunction with syndapin III to mediate caveolae formation in muscle tissue (Fra et al., 1995; Razani et al., 2002; Murata et al., 2007).

Over the past two decades, researchers have identified four caveolae coat-associated proteins with defined functions, namely, the cavin family (cavin 1–4). These cavins assemble into higher-order hetero-oligomeric structures, which are crucial for the architecture and functionality of caveolae. Cavin-1 (polymerase transcript release factor, PTRF) and cavin-2 (serum deprivation protein response, SDPR) are essential for caveolar formation and abundance, and they are the most extensively studied among these four proteins (Liu and Pilch, 2008; Tkachenko et al., 2012). Cavin-1 not only binds to phosphatidylinositol bisphosphate, but also associates with caveolar membrane through its interaction with phosphatidylserine (Busija et al., 2017). Both phosphatidylserine and phosphatidylinositol bisphosphate are necessary for caveolar formation. Moreover, Cavin-1 indirectly participates in caveolin-1 oligomerization by interacting with caveolin-1 in a 4:1 ratio (Ludwig et al., 2013). Cavin-1 knockout mice exhibit reduced caveolae and develop muscle hypertrophy through AKT signaling, as well as cardiac hypertrophy and fibrosis via extracellular regulated protein kinases (ERK) signaling (Taniguchi et al., 2016; Ding et al., 2017). Although cavin-2 is critical for the targeting of cavin-1 to the caveolar coat, its deletion does not have a widespread influence on caveolae throughout the body. For example, cavin-2 knockout reduced endothelial caveolae in the adipose tissue and lung tissue, but does not influence caveolae in the heart endothelium (Hansen et al., 2013). Moreover, cavin-2 affects caveolar membrane curvature, and its overexpression results in caveolae deformation and extensive plasma membrane tubulation (Hansen et al., 2009). *In vitro*, cavin-2 knockout leads to decreased endothelial cell proliferation, migration, angiogenesis, and nitric oxide production (Boopathy et al., 2017). Cavin-3 (srd-related gene product that binds to c-kinase, SRBC) knockout decreases caveolae density in smooth muscle cells, while having little effect on caveolae abundance in lung endothelial cells, adipocytes, and fibroblasts (Hansen et al., 2013; Liu et al., 2014; Zhu et al., 2017). Lastly, cavin-4 (muscle-restricted coiled-coil protein, MURC) is exclusively expressed in myocytes, and its overexpression increases caveolae abundance in cardiomyocytes *in vitro* (Ogata et al., 2014).

The hypothesis that caveolae-mediated transcytosis happens via vesicle transport and/or transient trans-endothelial pores formed by the fusion and fission of caveolae is supported by the integration of electron microscopy and cell biology studies (Peters et al., 1985; Ghitescu et al., 1986). Several laboratories have demonstrated the uptake of labeled plasma proteins into caveolar vesicles. Caveolae are typically identified using GFP-tagged caveolin-1 in live imaging studies. Evidence from total internal reflection fluorescence microscopy indicates that GFP-tagged caveolin-1 rapidly internalizes upon exposure to albumin in endothelial cells (Jiang et al., 2016; Zimnicka et al., 2016). Furthermore, the knockout of caveolin-1 in mice leads to decreased albumin internalization and reduced endothelial transcytosis (Schubert et al., 2001). In blood vessels, endothelial cells on the luminal side can transport lipids and proteins from the circulation to the abluminal side and *vice versa*. Additionally, other studies have confirmed that caveolae can transport insulin, albumin, and low-density lipoproteins (LDLs) (Vasile et al., 1983; Ghitescu et al., 1986; Nistor and Simionescu, 1986; Bendayan and Rasio, 1996). Thus, caveolae serve as the key mediators of transcellular transport within endothelial cells.

Other transient organelles, trans-endothelial channels, and vesicular-vacuolar organelles (VVOs) also play significant roles in fluid and solute permeability. VVOs are present in both tumor endothelium and normal venular endothelium, mediating the exudation of macromolecules in response to factors such as vascular endothelial growth factor (VEGF), serotonin, and histamine (Dvorak et al., 1996; Lin et al., 2008). Evidence from studies involving Caveolin-1 knockout mice indicates that caveolae are not essential for the formation of VVOs, suggesting that these organelles may be independent of caveolar structures (Chang et al., 2009). While VVOs have not been observed in capillaries, multiple studies have shown that the transcytotic transport of proteins with large molecular weight occur within capillary beds. Consequently, the question of whether VVOs are caveolae-independent structures or represent fused caveolar vesicles remains controversial. Additionally, transendothelial channels (TEC) are found in fenestrated endothelium, such as that in the exocrine pancreas, kidney cortex, and duodenal mucosa, but are absent in continuous endothelial cells (Stan, 2007).

### 3.2 Clathrin-mediated transcytosis

Clathrin is a highly evolutionarily conserved protein composed of a heavy chain with a molecular weight of 180 kDa and a light chain with a molecular weight ranging from 35 to 40 kDa, which together form a dimer. Three such dimers assemble to create the fundamental structural unit known as the triskelion (Kaksonen and Roux, 2018). Clathrin-mediated transcytosis is a vital process that facilitates the endocytosis of cargo through clathrin-coated pits and is observed across a range of cell types. Extensive research has identified multiple proteins participating in the initial phase of clathrin-coated vesicle formation at the cellular membrane (Kaksonen and Roux, 2018). For example, the adaptor complex adaptor protein 2 (AP2) promotes the formation of clathrin-coated pits consisting of clathrin light chain and heavy chain (Pearse, 1976; Motley et al., 2003). The GTPase dynamin triggers the detachment of pits from the plasma membrane, resulting in the formation of clathrin-coated vesicles (Sweitzer and Hinshaw, 1998). Subsequently, the clathrin coat is disassembled by the ATPase Heat shock cognate protein 70 (HSC70), and the uncoated vesicles then fuse with early endosomes, thereby entering the endosomal sorting pathway (Schlossman et al., 1984). Several receptors, including the insulin receptor and transferrin receptor, were found to experience clathrin-mediated endocytosis (Duffy and Pardridge, 1987; Roberts et al., 1993). Hyperinsulinemia has been shown to disrupt the internalization of insulin receptors into brain endothelial cells, impacting the regulation of molecular targets involved in cerebral blood flow (DiLucia et al., 2023). Recent studies have demonstrated that the transport of amyloid-beta peptide (Aβ) from the brain to the circulation relies on clathrin-mediated endocytosis (Deane et al., 2004; Zhao et al., 2015). Mechanistically, Aβ binds to the low-density lipoprotein receptor-Related Protein 1 (LRP1) receptor located on the abluminal side of the BBB, and the endocytic protein phosphatidylinositol binding clathrin-assembly protein (PICALM) is involved in regulating the clathrin-dependent internalization of the Aβ-LRP1 complex which leads to the endocytic transport and subsequent clearance of Aβ. Parvez Alam et al. observed significant colocalization of α-synuclein with clathrin, indicating that α-synuclein is internalized in brain endothelial cells via a clathrin-dependent mechanism (Alam et al., 2022). While α-synuclein did not colocalize with caveolin-1, the knockout of caveolin-1 in endothelial cells inhibits luminal to abluminal α-synuclein transcytosis, suggesting an indirect involvement of caveolin-1 in α-synuclein trafficking (Alam et al., 2022).

### 3.3 Macropinocytosis

Macropinocytosis is a nonselective endocytic pathway initiated by actin polymerization at the cell plasma membrane, which promotes the formation of membrane ruffles (Lim and Gleeson, 2011). These membrane ruffles represent extensions of the plasma membrane, and they may come from the leading edge of cells and then fold back, or originate from the dorsal surface of cells and then form the circular cups (Lim et al., 2015). Ruffles can encapsulate solutes into irregularly shaped vesicles, known as macropinosomes, which vary in size from 200 to 500 nm (Lim and Gleeson, 2011). Following their formation, macropinosomes can undergo homotypic fusion and fission and may also be transported to other organelles within the endosomal system (Wang et al., 2014). While early studies on macropinocytosis primarily focused on immune cells, such as dendritic cells and macrophages, subsequent research has identified this process in various non-immune cell types, such as neurons, epithelial cells, endothelial cell, fibroblasts, and cancer cells (West et al., 1989; Ridley et al., 1992; Wennström et al., 1994; Commisso et al., 2013; Holmes et al., 2013).

Macropinocytosis is associated with several biological functions, including cell metabolism, virus infection and gene expression. In human umbilical vein endothelial cells (HUVECs), glutamine deprivation enhances macropinocytosis, promoting the non-essential amino acids uptake and sustaining cell proliferation (Kim et al., 2017). In hCMEC/D3 cells, a type of human brain microvascular endothelial cells (HBMECs), the internalization of neutrophil-derived microvesicles alters the gene expression profile, leading to the dysregulation of genes related to vesicular transport, ubiquitin-mediated proteolysis, and tight junctions (Ajikumar et al., 2019). Various pharmacological inhibitors have been employed to elucidate the role of macropinocytosis in the uptake of neutrophil-derived microvesicles. These include cytochalasin D, which inhibits actin polymerization and microfilament formation; dynasore, which disrupts dynamin function; 5-(N-Ethyl-N-isopropyl) amiloride, which inhibits Na+/H+ exchangers crucial for macropinocytosis; genistein, which blocks caveolin-mediated endocytosis; and monodansylcadaverine, which inhibits clathrin-mediated endocytosis (Ajikumar et al., 2019). Additionally, macropinocytosis is also involved in viral entry into endothelial cells. For example, the bacterium *Escherichia coli* K1 enters HBMECs *via* micropinocytosis (Loh et al., 2017). Invasion of Kaposi’s sarcoma-associated herpesvirus (KSHV) into HUVECs and HBMECs is also *via* micropinocytosis. KSHV infection significantly elevates the membrane ruffling and uptake of 70 kDa dextran in the above 2 cell types (Raghu et al., 2009). Furthermore, macropinocytosis plays a critical role in the entry of human immunodeficiency virus 1 (HIV-1) into primary HBMECs, a process involving mitogen-activated protein kinase (MAPK) signaling, lipid rafts, and glycosaminoglycans (Liu et al., 2002).

Endothelial cells exhibit low levels of constitutive macropinocytosis under physiological conditions; however, this process can be upregulated in response to growth factors. In porcine aortic endothelial cells, basal membrane ruffling occurs at low levels. Platelet-derived growth factor (PDGF) enhances levels of PI(3,4,5)P3 and stimulates membrane ruffling, a process that requires phosphatidylinositol 3-kinase (PI3K) signaling (Wennström et al., 1994).

## 4 Methods for investigating transcytosis *in vitro* and *in vivo*

### 4.1 Assessment of transcytosis *in vitro*

**FIGURE 3:**
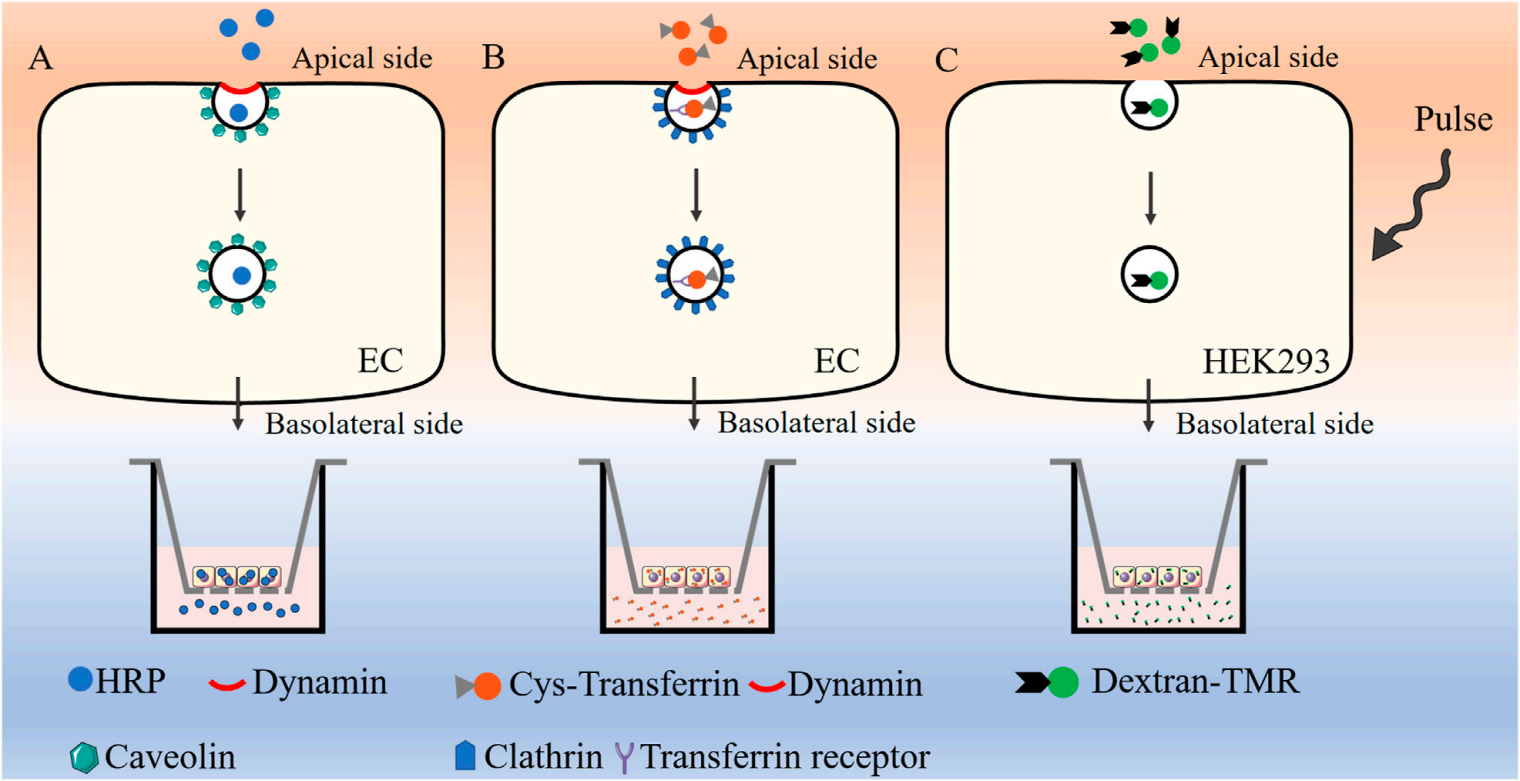
Assessment of transcytosis *in vitro*.HRP in conjunction with a fluorescent substrate **(A)**, Cy3-Tf **(B)**, or dextran-TMR **(C)** are used for the measurement of caveolae-mediated transcytosis, clathrin-mediated transcytosis, and macropinocytosis, respectively. HRP: Horseradish peroxidase, Cy3-Tf: Cyanine 3 labeled transferrin, dextran-TMR: dextran conjugated to tetramethylrhodamine, EC: Endothelial cell, HEK293: Human embryonic kidney 293 cells.

Transcytosis can be quantified via fluorescence-based assays conducted *in vitro*. In cell cultures, the application of horseradish peroxidase (HRP) in conjunction with a fluorescent substrate, fluorescently labeled transferrin (Cy3-Tf), or dextran conjugated to tetramethylrhodamine (dextran-TMR) allows for the precise measurement of caveolae-mediated transcytosis, clathrin-mediated transcytosis, and macropinocytosis events, respectively (Figure 3). These methodologies offer a robust and sensitive approach to elucidate the mechanisms underlying vesicle-mediated transport across the endothelial barrier, providing significant insights into the complex processes that govern transcytosis.

HRP-based assay is commonly used to evaluate caveolae-mediated transcytosis (Wang et al., 2020; Zhang et al., 2021) (Figure 3). When endothelial cells reach full confluency in the transwell chamber (TEER value around 20 Ω⋅cm^2^), they undergo serum starvation overnight at 37°C prior to the assay. A 2.5 mg/mL solution of HRP was added to the apical compartment of the epithelial layer and cells are incubated at 37°C for 20 min. The chambers are subsequently washed multiple times with precooled phosphate-buffered saline (PBS) to remove any unbound extracellular HRP. Once the washing steps were completed, fresh medium is added to both the upper and lower chambers, and incubation continues for an additional 60 min at 37°C. Following this incubation period, the medium from the lower chamber was collected, and HRP concentration was subsequently analyzed.

The transport of Cy3-Tf can be used to evaluate clathrin-mediated transcytosis in endothelial cells (Sade et al., 2014) (Figure 3). Endothelial cells were cultured to full confluency on transwell chamber and subjected to serum starvation overnight at 37°C before the assay began. The cells were then incubated apically with Cy3-Tf for 60 min at 37°C. Following incubation, the chambers were thoroughly washed several times with precooled PBS to remove any unbound extracellular Cy3-Tf. New medium was then added to both the upper and lower chambers, and the incubation continued for 60 min to release the endocytosed Cy3-Tf. The medium from the lower chamber was collected, and the corresponding fluorescence intensity of Cy3-Tf was measured.

Previous studies about assessment of macropinocytosis mainly focused on antigen-presenting cells such as macrophages and dendritic cells. David Liebl et al. reported a modified quantitative protocol to assess the rate and volume of macropinocytosis in human embryonic kidney cells (HEK293) (Figure 3), which could be also be extended to the measurement of micropinocytosis in other adherent cells (Bora et al., 2022). 10,000 MW dextran conjugated to tetramethylrhodamine was chose to measure micropinocytosis. HEK293 cells were cultured on poly-l-lysine-coated coverslips and subjected to serum starvation at least for 16 h before the assay. The cells were then pulsed with 100 μg/mL dextran–TMR for 5 min. The number of macropinosomes was quantified using automated image analysis. Future research is required to evaluate the practicality of this approach for analyzing endothelial macropinosomes.

### 4.2 Assessment of transcytosis *in vivo*

Current *in vivo* assessments of vascular leakage typically utilize colorimetric (such as HRP substrate and Evans Blue dye), fluorescent (dextran and sodium fluorescein), or radioactive tracers to detect extravasation from retinal vessels into adjacent tissues (Saunders et al., 2015). The ideal properties of a tracer for quantifying vascular leakage include inertness and large molecular size, which allow it to permeate damaged vessels and remain restricted within healthy ones. Evans blue dye binds with endogenous albumin to form a large molecule in the circulation after injection through the tail vein or intracardially, which could be visualized by fluorescence microscopy or quantified by spectrophotometric detection (Wu et al., 2020; Honeycutt and O'Brien, 2021; Li et al., 2023). Fluorescein isothiocyanate-conjugated dextran (FITC-dextran) and sodium fluorescein are wildly used in the assessment of retinal vascular leakage *in vivo* or *ex vivo* (Atkinson et al., 1991; Natarajan et al., 2017; Comin et al., 2021). Usually, 4 to 70 kD FITC-dextran can be selected based on specific experimental requirements. Additionally, FITC-albumin (approximately 68 kDa) serves as an alternative large-sized protein tracer for measuring vascular leakage (Pietra and Johns, 1996; Doggett and Breslin, 2014). Importantly, while these methods effectively detect retinal vascular leakage, they do not differentiate between trans-endothelial transport and paracellular transport. Specific tracers, such as HRP, are often utilized to locate trans-endothelial vesicles and analyze their ultra-microstructure under electron microscopy (Chow and Gu, 2017; Wang et al., 2020; Zhang et al., 2021).

Notably, vascular leakage assays, such as Evans Blue, FITC-dextran and albumin tracers cannot directly distinguish transcytosis from paracellular leakage. However, the methodologies described can indirectly infer transcytosis activity under specific experimental conditions, particularly when combined with complementary approaches, such as tracer size selection, electron microscopy validation and cell-specific pathway inhibition. First, transcytosis is typically associated with the transport of large molecules, while tight junctional dysfunction leads specifically to t the leakage of low-molecular-weight tracers 1 kDa and below (O'Brown et al., 2019). By comparing leakage of small tracers and large tracers under the same conditions, an increased permeability to large molecules may suggest active transcytosis rather than passive paracellular flux (Cui et al., 2021). Second, HRP tracers combined with ultrastructural analysis can visualize trans-endothelial vesicles and their cargo content. This approach provides direct evidence of transcytosis (Sadeghian et al., 2018). Third, pharmacological or genetic tools can block transcytosis without affecting paracellular junctions. Suppressed leakage of large tracer after such interventions could further support transcytosis involvement (Deffebach et al., 1996). Current methodologies do not fully resolve transcytosis-specific signals. In future work, dual-tracer imaging and endothelial-specific genetic models may help the study of transcytosis.

## 5 Transcytosis in pathological breakdown of the BRB

### 5.1 Plasmalemma vesicle-associated proteins

Endothelial cell-specific expression of PLVAP decreases during development and is absent once the functional BRB is established. The lack of PLVAP in barrier endothelial cells is crucial for both the formation and maintenance of the BRB (Daneman et al., 2010; Liebner and Plate, 2010; Umans et al., 2017). PLVAP is significant not only for preserving the fundamental vascular barrier function in particular subsets of vascular endothelial cells but also for contributing to BRB breakdown under pathological conditions, such as DR, AMD and ROP (Schlingemann et al., 1999; Witmer et al., 2002; Wisniewska-Kruk et al., 2016; Nakagami et al., 2019).

**FIGURE 4:**
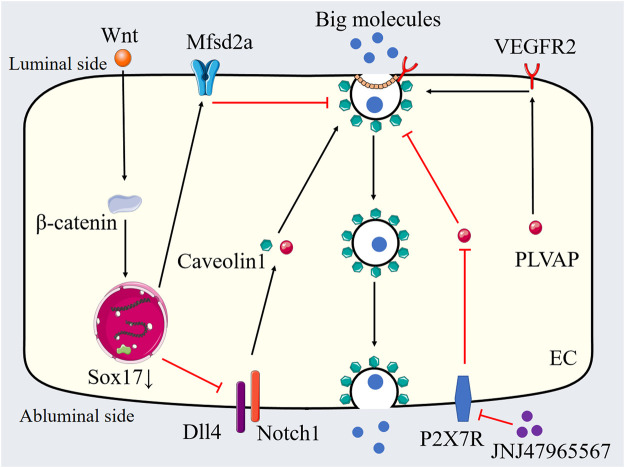
Transcytosis in pathological breakdown of the BRB. PLVAP, Wnt-Mfsd2a signaling and Dll4-Notch1 signaling are involved in the regulation of transcytosis in pathological breakdown of the BRB. JNJ47965567 inhibits transcytosis through P2X7R blockade-mediated regulation of PLVAP expression. PLVAP: Plasmalemma vesicle-associated proteins, Mfsd2a: Major facilitator superfamily domain containing 2a, Dll4: Delta-like ligand 4, VEGFR2: Vascular endothelial growth factor receptor 2, SOX7: SRY related high mobility group box 7, Wnt: Wingless-related integration site, P2X7R: Purinergic 2X7 receptor, EC: Endothelial cell.

VEGF has been proved to mediate several transcellular transport-associated genes and to increase caveolae-mediated transcytosis *in vivo*. Wisniewska-Kruk et al. demonstrated that the expression of PLVAP correlates with the loss of BRB properties in retinal capillaries, contributing to retinal vascular permeability in diabetic macular edema (DME). The knockdown of PLVAP reduced VEGF-mediated caveolae formation and the permeability of the BRB *in vitro* (Wisniewska-Kruk et al., 2016) (Figure 4). Different-sized tracers were utilized to explore the function of PLVAP inhibition in retinal vascular leakage in an *in vitro* BRB model. Previous studies indicate that larger molecules primarily penetrate the endothelial barrier through the transcellular pathway, while smaller molecules utilize the paracellular pathway. PLVAP inhibition decreased the permeability of 70 kDa tracers mediated by VEGF without affecting the permeability of 766 Da tracers (Wisniewska-Kruk et al., 2012; Wisniewska-Kruk et al., 2016). Furthermore, PLVAP inhibition did not influence VEGF-mediated changes in endothelial junctions (Wisniewska-Kruk et al., 2016). In an oxygen-induced retinopathy (OIR) mouse model, PLVAP inhibition was found to preserve BRB integrity, as indicated by reduced extravasation of 70 kDa fluorescent tracers and findings from fluorescein angiography (Wisniewska-Kruk et al., 2016). Therefore, PLVAP expression serves as a critical modulator of VEGF-induced transcellular transport. Notably, while PLVAP inhibition reduced VEGF-mediated caveolae formation to baseline levels, it did not affect the overall number of basal caveolae (Herrnberger et al., 2012; Wisniewska-Kruk et al., 2016). There was no significant decrease in the basal caveolae number in endothelial cells from PLVAP knockout mice. Although PLVAP inhibition does not regulate basal caveolae numbers under normal conditions, it undeniably plays a role in pathological vasogenic edema (Herrnberger et al., 2012; Wisniewska-Kruk et al., 2016). Given that vesicular trafficking is a complex biological process involving multiple proteins and molecules, changes in caveolae numbers may not necessarily correlate with alterations in transcytosis. However, the precise molecular mechanism by which PLVAP regulates caveolae formation and modulates cellular barrier function remains incompletely understood. Previous studies have shown that VEGF receptor 2 (VEGFR2) is localized in caveolae and that PLVAP expression is induced in a VEGFR2-dependent manner (Labrecque et al., 2003; Tahir et al., 2009). It is possible that PLVAP modulates VEGFR2 function to regulate caveolae formation and vascular barrier function. Notably, PLVAP has been found to interact with neuropilin-1 (NRP-1), an important co-receptor of VEGFR2 that regulates its surface expression (Keuschnigg et al., 2012; Gelfand et al., 2014). Therefore, PLVAP may influence the function or stability of VEGFR2/NRP-1 complexes, thereby modulating downstream VEGFR2 signaling. Further research is essential to clarify the precise molecular mechanisms involved in these processes. Besides VEGF, several signaling pathways and biological processes have been reported to modulate PLVAP expression, such as angiotensin-2, Norrin/wingless-related integration site (Wnt) signaling, transforming growth factor-β, Notch signaling, shear stress and tumor necrosis factor-α (Wasserman et al., 2002; Liebner et al., 2008; Liebner and Plate, 2010; Bodor et al., 2012; Farber et al., 2018).

Currently, anti-VEGF therapy is the primary treatment for DME. However, repeated intravitreal injections of anti-VEGF agents can lead to retinal fibrosis in DME patients with severe proliferative diabetic retinopathy (PDR) (Van Geest et al., 2012). Moreover, such injections have also been found to increase vascular leakage and induce retinal neurodegeneration in mouse models (Hombrebueno et al., 2015). Long-term anti-VEGF therapy may result in retinal damage, ultimately adversely affecting visual function. A recent study demonstrated that JNJ47965567 preserves BRB integrity through purinergic 2X7 receptor (P2X7R) blockade-mediated regulation of PLVAP expression (Platania et al., 2025). Notably, PLVAP is exclusively expressed in endothelial cells, suggesting that targeting PLVAP expression in retinal and brain endothelial cells potentially inhibit downstream VEGF signaling pathways without compromising neuronal cell survival. Additionally, PLVAP inhibition has been shown to significantly reduce retinal vascular leakage *in vivo* and protect cellular barrier function *in vitro*, indicating that anti-PLVAP treatment may represent a safer therapeutic option for DME.

### 5.2 Wnt-Mfsd2a signaling

Mfsd2a was initially identified as a regulator of BBB formation through the inhibition of transcytosis (Ben-Zvi et al., 2014). Although Mfsd2a knockout mice exhibited functional tight junctions, their BBB remained leaky due to increased transcytosis (Ben-Zvi et al., 2014). Brian Wai Chow et al. study the role of Mfsd2a in BRB function. Mfsd2a knockout resulted in elevated HRP-filled vesicle density in retinal endothelial cells of HRP-injected adult mice, without affecting tight junction integrity. Subsequent studies have shown that the active suppression of transcytosis is a fundamental mechanism regulating BBB function, which is dynamically influenced by various pathophysiological conditions. Our recent study provided direct evidence for the Mfsd2a-mediated regulation of transcytosis in diabetic retinal endothelial cells using the streptozotocin (STZ) model (Zhang et al., 2021). We demonstrated that decreased expression of endothelial Mfsd2a was associated with diabetic retinal vascular leakage. Transmission electron microscopy and HRP-based *in vitro* transcytosis assays revealed that downregulation of Mfsd2a led to increased endothelial vesicular transcytosis (Figure 4). We investigated four potential vesicle-trafficking-related proteins (caveolin1, SEC22 Homolog B (Sec22b), Syntaxin7 (STX7), and Slit-Robo Rho GTPase activating protein 2 (Srgp2)) in the STZ model, which have been reported to be regulated by Mfsd2a, and found that Sec22b is essential for endothelial vesicular transcytosis in the STZ model (Zhang et al., 2021). Overexpression of Mfsd2a reduced both type I trans-endothelial vesicles (vesicles forming *via* endocytosis from the luminal membrane) and type II trans-endothelial vesicles (already formed vesicles transported within the cytoplasm), thereby decreasing diabetic retinal vascular leakage. Furthermore, the combination of Mfsd2a overexpression and oral administration of DHA further reduced the density of type I trans-endothelial vesicles in the STZ mode (Zhang et al., 2021). Interestingly, another study found that the high dose fish oil treatment (818.2 mg/kg of eicosapentaenoic acid (EPA) and 545.5 mg/kg DHA per day, lasting for 21 days) elevated the expression of Mfsd2a without affecting DHA homeostasis in the retina and retinal pigment epithelium from healthy mice (Jovanović Macura et al., 2022). Another study found that High-dose fish oil supplementation increased Mfsd2a, and reducing AQP4 expression and Aβ deposition in retinal vessels of 5xFAD mice (Alzheimer’s mouse models), thereby suppressing transcytosis or enhancing lymphatic system function in the retina (Jovanovic Macura et al., 2024). These findings suggest that Mfsd2a expression may vary based on different pathophysiological conditions or DHA dosages, highlighting its critical role in mediating excessive endothelial transcytosis associated with retinal vascular leakage. Zhang et al. demonstrated that dysregulated transcytosis contributes to BRB breakdown in glaucoma pathogenesis. Their study revealed vascular leakage originating from the peripheral veins of the retinal ganglion cell layer, accompanied by significant downregulation of Mfsd2a. Notably, this pathological leakage occurred in the absence of endothelial junction disruption. The stabilization of β-catenin in retinal endothelial cells effectively prevented both BRB dysfunction and subsequent neurodegeneration in the glaucoma murine model (Zhang et al., 2024).

Wang et al. explored the role of Wnt signaling in retinal endothelial cell transcytosis and BRB function in 2 mouse models: low-density lipoprotein receptor–related protein 5 (Wnt co-receptor) knockout (Lrp5^−/−^) mice and Norrin (Wnt ligand) knockout (Ndp^y/−^) mice. Both models showed elevated retinal endothelial transcytosis and increased retinal vascular leakage (Wang et al., 2020). Wnt signaling was found to directly regulate the expression of Mfsd2a in a β-catenin–dependent manner, Mfsd2a overexpression inhibited Wnt deficiency–mediated transcytosis in endothelial cells (Figure 4). Additionally, both models displayed elevated caveolin-1 expression and an abundance of caveolar vesicles. Mfsd2a overexpression led to a reduction in caveolin-1 levels, as well as a reduction in type I and type II trans-endothelial vesicles and the density of immunogold-labeled caveolin-1–positive trans-endothelial vesicles in Lrp5^−/−^ mice. The research suggests that Wnt signaling plays a crucial role in mediating Mfsd2a-dependent transcytosis within the vascular endothelium, specifically through a caveolin-1–positive caveolae pathway (Wang et al., 2020). Furthermore, the loss of Apcdd1, which acts as a negative regulator of Wnt signaling, increased the transcriptional activity of endothelial β-catenin, leading to delayed vessel pruning and premature BRB formation in the postnatal retina (Mazzoni et al., 2017). *In vitro* studies on Apcdd1^−/−^ brain endothelial cells revealed an increase in transcytosis and a compromised transcellular barrier, which could be partially reversed by exposure to Wnt3a. Interestingly, there were no notable differences in PLVAP and caveolin-1 protein expression or albumin uptake between Apcdd1 mutant mice and their wild-type littermate controls. The authors hypothesized that the loss of Apcdd1 in brain endothelial cells might promote the maintenance of PLVAP-expressing fenestrae, leading to an increase in albumin uptake. This phenomenon may be attenuated *in vivo* due to pericyte-derived signals that could reduce PLVAP expression in Apcdd1^−/−^ retinal endothelial cells (Mazzoni et al., 2017).

### 5.3 Dll4-Notch1 signaling

Delta-like ligand 4 (Dll4) plays a multifaceted role in the development of the vascular system, with its functions in adult vasculature, particularly in lacteal and cardiac vessels, becoming increasingly recognized (Hellström et al., 2007; Suchting et al., 2007; Bernier-Latmani et al., 2015; Pitulescu et al., 2017; Jabs et al., 2018). Jee Myung Yang et al. investigated Dll4’s role in maintaining the integrity of the BRB (Yang et al., 2020). Their study initially examined the function of Dll4 in BRB maintenance in adult mice. A single intravitreal injection of anti-Dll4 antibody caused the extravasation of dextran (4 and 10 kDa) and Evans blue dye in the superficial vascular plexus. Leakage of Evans blue from arteries was detected 3 days post-injection, while leakage in veins and capillaries was observed after 7 days (Yang et al., 2020). By 10 days, Evans blue leakage diminished and became restricted to periarterial regions. Further research indicated that endothelial Dll4-Notch1 signaling is crucial for maintaining retinal barrier integrity by inhibiting transcytosis. Genetic or antibody-mediated inactivation of Dll4 resulted in increased immunostaining of caveolin-1 and PLVAPs in retinal arteries and arterioles, without altering the levels or distribution of VE-cadherin, zonula occludens-1 (ZO-1), claudin-5, or occludin (Figure 4) (Yang et al., 2020). Additionally, inhibition of Notch1 led to the extravasation of tracers and elevated expressions of caveolin-1 and PLVAPs in endothelial cells, while not affecting VE-cadherin and claudin-5 expression. The SRY-box transcription factor 17 (Sox17) serves as an upstream regulator of Dll4; endothelial knockout of Sox17 reduced arterial Dll4 expression and led to tracer extravasation, increased PLVAP and caveolin-1 expression, and a rise in transcellular vesicles in arterial endothelial cells (Yang et al., 2020) (Figure 4). Furthermore, sterol regulatory element-binding protein 1 (SREBP1) mediates Dll4 inhibition-induced transcytosis. The SREBP1 inhibitor fatostatin reduced dextran leakage, the expression of PLVAP, and the formation of transcellular vesicles following the intravitreal injection of anti-Dll4 antibody (Yang et al., 2020). In a model of hypertension-induced retinal edema, the Sox17-Dll4-SREBP1 signaling axis was also implicated in pathological retinal vascular leakage (Yang et al., 2020).

## 6 Targeting transcytosis for drug delivery across the BRB

The BRB comprises the retinal capillary endothelial cells (RCECs) and the RPEs. RCECs establish the inner blood-retinal barrier, while RPEs establish the outer barrier. Both cell types feature tight junctions that restrict the passage of drugs between the neural retina and the circulating blood. Within the RPEs, specialized transport proteins and vesicles facilitate the exchange of specific active pharmaceuticals between the choroid and the retina. Strategies for drug delivery to the retina typically involve creating complexes between drugs and receptor-targeting entities or directing them to specific transport vesicles.

Previous research has demonstrated that clathrin-mediated endocytosis, caveolae-mediated endocytosis, and macropinocytosis can operate independently or synergistically to mediate drug delivery. For instance, iron delivery to the CNS occurs through the specific binding and intracellular trafficking of transferrin, and the transferrin receptor has been shown to be a key target for retinal drug delivery. Atsushi Imakiire et al. identified a fusion protein, pabinafusp alfa, which consists of iduronate-2-sulfatase and an antihuman transferrin receptor antibody. This fusion protein demonstrated the ability to cross the BRB and effectively reached retinal tissue in a murine model of mucopolysaccharidosis (Imakiire et al., 2023). Rathapon Asasutjarit et al. developed a transferrin-conjugated liposomes containing ganciclovir (Tf-GCV-LPs), which can be administered *via* intravitreal injection or topical instillation. Tf-GCV-LPs exhibited a negative zeta potential, a particle size of less than 100 nm, and good biosafety in ARPE-19 cells. These liposomes were internalized by ARPE-19 cells through transferrin receptor-mediated endocytosis and exhibited inhibitory activity against cytomegalovirus in infected cells (Asasutjarit et al., 2020). Moreover, functionalizing the surface of dual-functionalized poly (lactide-co-glycolide) nanoparticles with transferrin, a linear arginine-glycine-aspartic acid peptide, or a combination of both enhanced the retinal delivery of nanoparticles following intravenous administration (Singh et al., 2009). These findings support the hypothesis that transferrin receptor-conjugated drugs can penetrate the BRB. G. Puras developed a novel niosome formulation utilizing the cationic lipid 2,3-di(tetradecyloxy)propan-1-amine in combination with squalene and polysorbate 80 (Puras et al., 2014). The lipoplexes, prepared at a 15:1 ratio (cationic lipid/DNA), displayed a zeta potential of 25 mV, a size of 200 nm, and spherical morphology, effectively protecting DNA from enzymatic degradation. These lipoplexes predominantly entered ARPE-19 cells *via* clathrin-mediated endocytosis, while HEK-293 cells showed a preference for caveolae-dependent entry (Puras et al., 2014). Zhang et al. successfully prepared pure and active Tat PTD-endostatin from *E. coli*, demonstrating anti-angiogenic activity both *in vivo* and *in vitro* (Zhang et al., 2015). *In vitro* experiments indicated that the uptake of Tat PTD-endostatin primarily occurred *via* endocytosis, with both clathrin-mediated and caveolae-mediated endocytosis contributing to its internalization. Tat PTD-endostatin administered as eye drops effectively crossed ocular barriers to reach the retina (Zhang et al., 2015). Mohamed Mashal designed a novel formulation based on the *N*-[1-(2,3-dioleoyloxy)propyl]-*N*,*N*,*N*-trimethylammonium chloride (DOTMA) cationic lipid and the polysorbate 60 non-ionic surfactant for the delivery of genetic material into the retina (Mashal et al., 2017). Niosomes containing lycopene exhibited a positive zeta potential, nanometric size, and low polydispersity, successfully transfecting the outer segments of the rat retina following subretinal or intravitreal administration. *In vitro* assays indicated that caveolae-mediated endocytosis and micropinocytosis were the likely internalization pathways for these niosomes (Mashal et al., 2017). Additionally, niosomes formulated with squalene as a helper lipid demonstrated high transfection efficiency in ARPE-19 cells, largely due to significant cellular uptake and the utilization of specific entry pathways, particularly macropinocytosis and lysosomal escape mechanisms (Ojeda et al., 2016). Stearylated (STR)-CH2R4H2C (CH2R4), a cytoplasm-responsive STR peptide, facilitates stable siRNA complexation, enhances cell permeation, and enables the control of intracellular dynamics. The cationic amino acids, particularly arginine, in CH2R4 promote cellular uptake *via* macropinocytosis by activating cytoskeletal F-actin, while its cysteine residue contributes to siRNA release (Nishida et al., 2023). Li et al. developed micellar dasatinib that encapsulated dasatinib into nanoparticles prepared from micellation of PEG-b-PCL. Micellar dasatinib significantly suppressed cell adhesion, migration, and proliferation compared to free dasatinib, likely due to enhanced solubility (Li et al., 2016). *In vitro* assays indicated that micellar dasatinib entered ARPE-19 cells *via* both caveolae-mediated and clathrin-mediated endocytosis. Furthermore, human serum albumin nanoparticles (HSA NPs) demonstrate significant potential as gene delivery vectors for effective gene therapy applications, with cellular uptake mediated by both clathrin-dependent and caveolae-dependent endocytosis (Mo et al., 2007). Current advancements in adeno-associated virus (AAV)-based gene therapy have demonstrated robust clinical and preclinical success for inherited retinal diseases. The AAV serotypes 1, 2, 4, 5, 7, 8, and 9 exhibit efficient transduction of RPE cells, while photoreceptor transduction efficacy varies significantly across serotypes. Notably, chimeric variants AAV2/5, 2/7, 2/8, and 2/9 show superior photoreceptor-targeting capabilities. However, the mechanisms underlying vector traversal across the BRB remain unclear. Ding et al. revealed that AAV2 traverse RPE cells *via* transcytosis to reach photoreceptors following suprachoroidal administration. This transport process subjected the vectors to proteasomal degradation, inhibition of which significantly enhanced photoreceptor transduction efficiency (Ding et al., 2021). Recently, Wang et al. developed a vascular cell adhesion molecule 1 (VCAM1)-targeted ultrasound-activated liposomal system capable of recognizing VCAM1 on vascular endothelial cells, initiating receptor-mediated transcytosis to achieve transendothelial transport towards biofilm-associated surgical site infection areas. However, the feasibility of this approach for penetrating the BRB requires further experimental validation (Wang et al., 2025).

Mfsd2a is a promising target for drug delivery across the BRB. Two primary Mfsd2a-based strategies exist for facilitating the transport of drugs from the blood to the retina (Wang et al., 2016). First, the reversible suppression of Mfsd2a may lead to a temporary increase in transcytosis in retinal vascular endothelial cells, thereby enhancing the transport of macromolecular drugs across the BRB. Previous evidence indicates that Mfsd2a mediates the transport of tunicamycin across cellular barriers (Reiling et al., 2011). Notably, Ben-Zvi et al. proposed tunicamycin and its structural derivatives as potential Mfsd2a inhibitors, and Wang et al. hypothesized that tunicamycin-mediated suppression of Mfsd2a may be reversible, because transient binding occurs during active transport followed by subsequent dissociation within the central nervous system microenvironment (Wang et al., 2016). Further investigations are needed to optimize Mfsd2a inhibitor design through structural and functional characterization. Second, Mfsd2a can function as a transport mechanism for specific small-molecule drugs that are chemically conjugated to LPC for delivery across the BRB. Future research should focus on improving tissue specificity and drug uptake efficiency to enhance the effectiveness of transcytosis-mediated drug delivery systems.

## 7 Conclusion

Retinal endothelial transcytosis is a crucial process for transporting macromolecules from the vascular compartment into surrounding tissues. While the role of transcytosis in the development of the BRB and in diseases associated with BRB breakdown has been preliminarily studied, several significant unresolved issues warrant further investigation. For instance, the real-time dynamics of transcytosis in the retina remain unclear. Developing new methods to visualize and assess the dynamics of endothelial transcytosis *in vivo* is essential for understanding the directionality and speed of vesicular trafficking. Additionally, it is important to explore whether mechanisms beyond caveolae-mediated transcytosis, clathrin-mediated transcytosis, and micropinocytosis regulate this process. Furthermore, the neurovascular unit comprises various cell types and structures that collaborate to maintain retina homeostasis. It remains unclear whether the crosstalk between endothelial cells and other cell types affects endothelial transcytosis. Finally, significant challenges persist in developing efficient and specific drug delivery systems based on transcytosis. A comprehensive understanding of BRB transport, protein engineering, and pharmacokinetics is essential for improving the clinical applicability of transcytosis-mediated drug delivery systems.

## Glossary

Aβ: Amyloid-beta peptide
AJs: Adherens junctions
AMD: Age-related macular degeneration
AP2: Adaptor protein 2
APCDD1: Adenomatosis polyposis coli downregulated 1
BBB: Blood-brain barrier
BRB: Blood-retina barrier
CH2R4: CH2R4H2C
CNS: Central nervous system
Dextran-TMR: Dextran conjugated to tetramethylrhodamine
DHA: Docosahexaenoic acid
DLL4: Delta-like ligand 4
DME: Diabetic macular edema
DOTMA: N-[1-(2,3-dioleoyloxy)propyl]-N,N,N-trimethylammonium chloride
DR: Diabetic retinopathy
*E. coli* K1: *Escherichia coli* K1
EPA: Eicosapentaenoic Acid
ERK: Extracellular regulated protein kinases
FDs: Fenestral diaphragms
FITC: Fluorescein isothiocyanate
FITC-dextran: Fluorescein isothiocyanate-conjugated dextran
HBMECs: Human brain microvascular endothelial cells
HEK293: Human embryonic kidney 293 cells
HIV-1: Human immunodeficiency virus 1
HREC: Human retinal endothelial cells
HRP: Horseradish peroxidase
HSA NPs: Human serum albumin nanoparticles
HSC70: Heat shock cognate protein 70
HUVECs: Human umbilical vein endothelial cells
KSHV: Kaposi’s sarcoma-associated herpesvirus
LDLs: Low-density lipoproteins
LPC: Lysophosphatidylcholine
LRP1: Low-density lipoprotein receptor-related protein 1
LRP5: Low-density lipoprotein receptor–related protein 5
MAPK: mitogen-activated protein kinase
Mfsd2a: Major facilitator superfamily domain containing 2a
MURC: Muscle-restricted coiled-coil protein
NKCC: Sodium-potassium-two chloride (Na^+^-K^+^-2Cl^-^) cotransporter
Ndp: Norrin
NRP-1: Neuropilin-1
OIR: Oxygen-induced retinopathy
P2X7R: Purinergic 2X7 receptor
PDGF: Platelet-derived growth factor
PDR: Proliferative diabetic retinopathy
PI3K: Phosphatidylinositol 3-kinase
PICALM: Phosphatidylinositol binding clathrin-assembly protein
PLVAP: Plasmalemma vesicle-associated protein
PTRF: Polymerase transcript release factor
RCECs: Retinal capillary endothelial cells
RMECs: Retinal microvascular endothelial cells
ROP: Retinopathy of prematurity
RPEs: Retinal pigment epithelial cells
SDPR: Serum deprivation protein response
SDs: Stomatal diaphragms
Sec22b: SEC22 Homolog B
SOX17: SRY-box transcription factor 17
SRBC: Srd-related gene product that binds to C-kinase
SREBP1: Sterol regulatory element-binding protein one
Srgp2: Slit-Robo Rho GTPase activating protein two
STR: Stearylated
STX7: Syntaxin7
STZ: Streptozotocin
TEC: Transendothelial channels
Tf-GCV-LPs: Transferrin-conjugated liposomes containing ganciclovir
TJs: Tight junctions
VCAM1: vascular cell adhesion molecule one
VEGF: Vascular endothelial growth factor
VEGFR2: Vascular endothelial growth factor receptor two
VVOs: Vesicular-vacuolar organelles
Wnt: Wingless-related integration site
ZO-1: Zonula occludens-1.
